# Osseous Union of Teeth

**Published:** 1853-10

**Authors:** M. K. Bridgeman


### ARTICLE V.

*Osseous Union of Teeth.* By M. K. Bridgeman.

The perusal of an article on the *osseous union of teeth*, page 333, of your number for January, of the present year, reminded me of a similar case which came under my notice some eight or nine years since. In this instance, it occurred with the *permanent* teeth of the *lower* jaw—the right central being united with the right lateral incisor.

The patient, a youth twelve years of age, was brought to me to have my opinion as to the propriety of removing the *left cuspid* on account of its projecting outwards; but as the sacrifice of this tooth was quite out of the question when the absence of the *left central incisor* would have made the mouth all that could have been desired in respect of form. This, however, was not consented to, and I have not seen the mouth since.

There appeared to be a strong superstitious feeling on the part of the mother with regard to the tooth, or rather teeth, in question; and although I ultimately obtained her consent to allow an impression to be taken, it was most reluctantly accorded, and was evidently so understood by the lad himself, for his unwillingness was little short of being positively refractory.

As I have not the power of sending you the *original*, I forward its most valuable substitute, a plaster cast of it "in situ," and I much regret not having succeeded in obtaining a more perfect one, still, under these circumstances, I considered myself fortunate in getting one at all. The posterior side shows the line of junction more decidedly than the anterior, and at the lower portion of enamel just above the neck, it nearly disappears; for this I have no doubt but that there exists a less perceptible separation in the fang than the crown.

I enclose also a cast of an *upper right dens sapientiæ* with a small supernumerary tooth adjoining it. That it is really united to the tooth, I am quite positive; I convinced myself of this by passing a fine flat pointed probe between them, and by pressing the smaller one outward, both moved in concert.

*Norwich, England.*

